# Clinical impact of cardiac magnetic resonance in patients with suspected coronary artery disease associated with chronic kidney disease (AQUAMARINE-CKD study): study protocol for a randomized controlled trial

**DOI:** 10.1186/s13063-022-06820-w

**Published:** 2022-10-24

**Authors:** Teruo Noguchi, Hideki Ota, Naoya Matsumoto, Yoshiaki Morita, Akira Oshita, Eiji Kawasaki, Tomohiro Kawasaki, Kensuke Moriwaki, Shingo Kato, Kazuki Fukui, Tomoya Hoshi, Hiroaki Watabe, Tomoaki Kanaya, Yasuhide Asaumi, Yu Kataoka, Fumiyuki Otsuka, Kensuke Takagi, Shuichi Yoneda, Kenichiro Sawada, Takamasa Iwai, Hideo Matama, Satoshi Honda, Masashi Fujino, Hiroyuki Miura, Kunihiro Nishimura, Kei Takase

**Affiliations:** 1 Department of Cardiovascular Medicine, National Cerebral and Cardiovascular Center, 6-1 Kishibe-shimmachi, Suita, 564-8565 Japan; 2 Department of Diagnostic Radiology, Tohoku University Hospital, 1-1 Seiryo-machi, Aoba-ku, Sendai, 980-8574 Japan; 3 Division of Cardiology, Department of Medicine, Nihon University School of Medicine, Nihon University Hospital, 1-6 Kanda-surugadai, Chiyoda-ku, Tokyo, 101-8309 Japan; 4 Department of Radiology, National Cerebral and Cardiovascular Center, 6-1 Kishibe-Shimmachi, Suita, 564-8565 Japan; 5 Department of Cardiology, Ehime Prefectural Imabari Hospital, 4-5-5 Ishiicho, Imabari, 794-0006 Japan; 6 Department of Diabetes and Endocrinology, Shin-Koga Hospital, 120 Tenjin-cho, Kurume, 830-8577 Japan; 7 Cardiovascular and Heart Rhythm Center, Shin-Koga Hospital, 120 Tenjin-cho, Kurume, 830-8577 Japan; 8 Comprehensive Unit for Health Economic Evidence Review and Decision Support, Research Organization of Science and Technology, Ritsumeikan University, Kyoto, 604-8520 Japan; 9 Department of Cardiovascular Medicine, Kanagawa Cardiovascular and Respiratory Center, Yokohama, 236-0051 Japan; 10 Cardiovascular Division, Faculty of Medicine, University of Tsukuba, Ibaraki, 305-8576 Japan; 11 Department of Cardiovascular Medicine, Dokkyo Medical University Hospital, 880 kitakobayashi, Mibu, Tochigi, 321-0293 Japan; 12 Department of Preventive Medicine and Epidemiology, National Cerebral and Cardiovascular Center, 6-1 Kishibe-Shimmachi, Suita, 564-8565 Japan; 13 Department of Diagnostic Radiology, Tohoku University Graduate School of Medicine, 2-1 Seiryo-machi, Aoba-ku, Sendai, 980-8575 Japan

**Keywords:** Cardiac magnetic resonance, Coronary artery disease, Chronic kidney disease, Plaque imaging

## Abstract

**Background:**

Although screening for coronary artery disease (CAD) using computed tomography coronary angiography in patients with stable chest pain has been reported to be beneficial, patients with chronic kidney disease (CKD) might have limited benefit due to complications of contrast agent nephropathy and decreased diagnostic accuracy as a result of coronary artery calcifications. Cardiac magnetic resonance (CMR) has emerged as a novel imaging modality for detecting coronary stenosis and high-risk coronary plaques without contrast media that is not affected by coronary artery calcification. However, the clinical use of this technology has not been robustly evaluated.

**Methods:**

AQUAMARINE-CKD is an open parallel-group prospective multicenter randomized controlled trial of 524 patients with CKD at high risk for CAD estimated based on risk factor categories for a Japanese urban population (Suita score) recruited from 6 institutions. Participants will be randomized 1:1 to receive a CMR examination that includes non-contrast T1-weighted imaging and coronary magnetic angiography (CMR group) or standard examinations that include stress myocardial scintigraphy (control group). Randomization will be conducted using a web-based system. The primary outcome is a composite of cardiovascular events at 1 year after study examinations: all-cause death, death from CAD, nonfatal myocardial infarction, nonfatal ischemic stroke, and ischemia-driven unplanned coronary intervention (percutaneous coronary intervention or coronary bypass surgery).

**Discussion:**

If the combination of T1-weighted imaging and coronary magnetic angiography contributes to the risk assessment of CAD in patients with CKD, this study will have major clinical implications for the management of patients with CKD at high risk for CAD.

**Trial registration:**

Japan Registry of Clinical Trials (jRCT) 1,052,210,075. Registered on September 10, 2021.

**Supplementary Information:**

The online version contains supplementary material available at 10.1186/s13063-022-06820-w.

## Background

Computed tomography coronary angiography (CTCA) has been reported to be clinically beneficial in patients with stable chest pain due to coronary artery disease (CAD) [1, 2]. However, CTCA has disadvantages such as iodinated contrast agent use. In addition, the diagnostic accuracy of CTCA is limited by the amount of coronary artery calcification.

Coronary magnetic resonance angiography (CMRA) has emerged as an alternative imaging modality for visualizing coronary arteries [3, 4], and its prognostic value has been reported in patients with suspected CAD [5]. Moreover, the presence of high-intensity plaques (HIPs) detected with non-contrast T1-weighted imaging (T1WI) is associated with future coronary events [6]. Thus, the combination of whole-heart CMRA and non-contrast T1WI may be promising modalities for evaluating coronary artery stenosis and plaque characteristics simultaneously. However, no studies have investigated the clinical utility of these techniques for risk stratification and management of CAD. Moreover, no evaluations of the cost-effectiveness of CMRA plus non-contrast T1WI for investigating CAD have been performed to date.

Chronic kidney disease (CKD), defined as dysfunction of the glomerular filtration apparatus, is an independent risk factor for the development of CAD [7]. Although mortality risk in patients with CAD progressively increases with worsening CKD, the optimal imaging strategy for evaluating CAD in patients with CKD has been remained. Some patients with CKD have extensive calcification disproportionate to the severity of obstructive CAD [7, 8], which limits the diagnostic value of the CT calcium score. In addition, late-stage CKD has been associated with an increased risk of post-contrast acute kidney injury from CTCA. Thus, to date, myocardial perfusion scintigraphy and stress echocardiography can be used to assess obstructive CAD in patients with advanced CKD.

We will conduct a prospective multicenter randomized study called the Attempts at Plaque Vulnerability Quantification with Magnetic Resonance Imaging Using Non-contrast T1-weighted Technique (AQUAMARINE) [6]-CKD study to investigate the clinical impact of non-contrast T1WI and CMRA on management and risk stratification in patients with CKD and CAD or suspected CAD.

## Methods/design

### Aim

The aim of this study is to determine whether coronary HIPs visualized using non-contrast T1WI and coronary artery stenosis detected by CMRA can predict mortality and future coronary events in study participants receiving primary prevention for CAD.

### Study design

This is a multicenter, parallel-group, prospective randomized controlled, open-label, superiority trial to assess the impact of non-contrast T1WI and CMRA on the diagnosis and management of patients at high risk for CAD estimated based on CAD risk factor categories for a Japanese urban population (Suita score), which is composed of 8 coronary risk factor categories (Tables 1 and 2) [9, 10]. Eligible subjects are randomly assigned.

**Table 1:** Suita score

Risk factor	Value	Score
Age (years)	35–44	30
	45–54	38
	55–64	45
	65–69	51
	> 70	53
Gender	Female	− 7
Current smoking	Yes	5
Diabetes mellitus	Yes	6
Blood pressure (mmHg)	Optimal (SBP < 120 and DBP < 80)	− 7
	Normal (SBP < 130, DBP < 85)	0
	High normal (SBP < 140 or DBP < 90)	0
	Stage I HTN (SBP 140–159 or DBP 90–99)	4
	Stage II–IV HTN (SBP 160–179 or DBP 100–109)	6
LDL-cholesterol (mg/dl)	< 100	0
	100–139	5
	140–159	7
	160–179	10
		11
HDL-cholesterol (mg/dl)	< 40	0
	40–59	− 5
	≥ 60	− 6
CKD	Stage I or II (eGFR > 60 ml/min/1.73 m^2^)	0
	Stage III (eGFR: 30–59 ml/min/1.73 m^2^)	3
	Stage 4 or 5 (eGFR < 30 ml/min/1.73 m^2^)	14

Weighted scoring of risk factors is used to categorize the 10-year risk of coronary heart disease. *CHD* coronary heart disease, *CKD* chronic kidney disease, *eGFR* estimated glomerular filtration rate, *DBP* diastolic blood pressure, *HDL* high-density lipoprotein, *HTN* hypertension, *LDL* low-density lipoprotein, *SBP* systolic blood pressure

**Table 2:** Predicted probability of CHD in 10 years

Total Suita score	Estimated 10-year risk
< 35	< 1%
36–40	1%
41–45	2%
46–50	3%
51–55	5%
56–60	9%
61–65	14%
66–70	22%
> 71	> 28%

to the CMR group or the control group (allocation rate 1:1). The randomization stratified on the following variables: age, gender, presence or absence of type 2 diabetes mellitus, and CKD grade (Fig. 1).

**Fig. 1:**
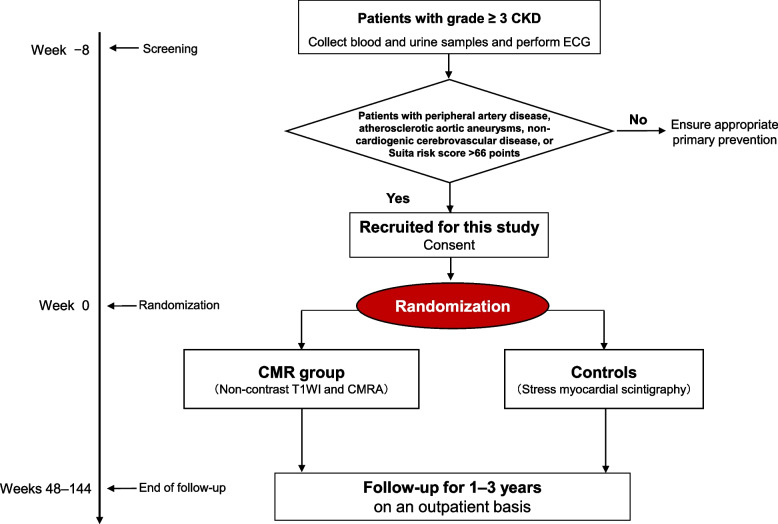
Trial design. CAD coronary artery disease, CKD chronic kidney disease, CMR cardiac magnetic resonance, CMRA coronary magnetic resonance angiography, ECG electrocardiogram, T1WI T1-weighted imaging

### Patient eligibility and recruitment

Patients aged 20 years or older with high cardiac risk estimated based on risk factor categories for a Japanese urban population (Suita score ≥66 points, corresponding to an estimated 10-year risk of CAD >22%) will be recruited from 6 institutions (Supplementary Figure 1). We will randomly assign 524 patients with CKD at high risk for CAD to evaluation with CMR (CMR group, 262 patients) at 6 institutions or to standard examinations that include stress myocardial scintigraphy (control group, 262 patients). Investigations, treatments, and clinical outcomes will be assessed over 1 year (short-term prognosis) and 3 years (long-term prognosis) (Fig. 1).

### Interventions

Eligible patients underwent non-contrast T1WI and CMRA (CMR group) or stress myocardial scintigraphy (control group) at each institution (Supplementary Figure 1). There is no anticipated harm and compensation for trial participation.

### Study procedures

**Fig. 2:**
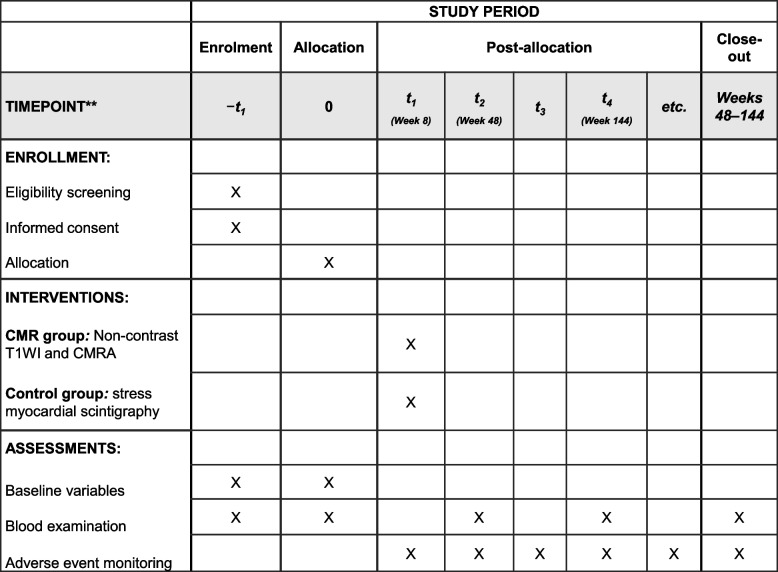
Sample timeline for enrollment, interventions, and assessments. CMR cardiac magnetic resonance, CMRA coronary magnetic resonance angiography, T1WI T1-weighted imaging

Eligible patients will be randomly assigned to the CMR group or the control group. After each examination, follow-up will be performed until 144 weeks after allocation (Fig. 2).

### Inclusion and exclusion criteria

#### Inclusion criteria

Study participants must meet all of the following criteria:

1. Age of 20–85 years
2. Diagnosis of hypertension, type 2 diabetes mellitus, non-cardiogenic cerebral infarction, peripheral arterial disease, carotid stenosis, aortic disease, or dyslipidemia
3. Grade ≥ 3 CKD with estimated glomerular filtration rate (eGFR) < 60 ml/min/1.73 m^2^ and very high risk for CAD, with a Suita score of ≥66 points with a predicted 10-year incidence of a coronary event of >22%
4. Capable of visiting a study center on an outpatient basis
5. Capable of providing written informed consent before study participation

#### Exclusion criteria

Individuals who meet any of the following criteria are ineligible for the study:

1. Suspected acute coronary syndrome on assignment
2. Acute myocardial infarction (MI) according to the 4th universal definition [11]
3. MI within 3 months prior to obtaining consent for study participation
4. History of percutaneous coronary intervention (PCI) with stenting or coronary artery bypass grafting (CABG)
5. Need for a coronary CT scan
6. Hemodialysis or peritoneal dialysis
7. MR unsafe devices in the body
8. History of cardiac surgery or thoracic surgery with sternotomy
9. Claustrophobia
10. Chronic atrial fibrillation
11. Frequent atrial or ventricular extrasystoles on 12-lead ECG at rest
12. Physician judgment that beta-blocker therapy would be inadvisable (e.g., due to arteriosclerosis obliterans with pain at rest, second-degree or higher atrioventricular block, sick sinus syndrome, or active bronchial asthma)
13. Pregnancy, lactation, or being female and unable to consent to contraception during study participation
14. Participation in other clinical studies or are scheduled to participate in other clinical studies during this study
15. Principal investigator or sub-investigator judgment that study participation would be inappropriate for other reasons
16. Patient desire to discontinue study participation or withdraw consent

All eligible patients will be invited to enter the trial by their attending physician. Written informed consent will be obtained from patients willing to participate in the study.

### Outcomes

#### Primary endpoint

The primary outcome is a composite of cardiovascular events at 1 year after study examinations: all-cause death, death from cardiovascular disease, nonfatal MI, nonfatal ischemic stroke, and ischemia-driven* unplanned coronary intervention (PCI or CABG).

*Myocardial ischemia diagnosed with stress myocardial single photon emission computing tomography (SPECT), or invasive fractional flow reserve.

#### Secondary endpoints

1. Incidence of composite cardiovascular events at 3 years after study examinations
2. Cost-effectiveness analysis
3. Management status (prescription rates of secondary prevention drugs for CAD and rates of achieving management goals for blood pressure, lipids, and diabetes), additional imaging tests (stress myocardial SPECT or 13 N-ammonia positron emission tomography [NH_3_-PET]), and PCI or CABG after study examinations
4. Rate of complications associated with coronary artery HIPs with a plaque-to-myocardium signal intensity ratio (PMR) of ≥ 1.1 or coronary artery HIPs with PMR ≥ 1.4 in patients at very high risk (Suita score of ≥66) and undergoing primary prevention for CAD
5. Complication rate for coronary artery HIPs by CKD grade

### CMR imaging

Non-contrast T1WI will be performed with a 3-T MR imager (MAGNETOM Verio; Siemens Healthineers, Erlangen, Germany) with a 32-channel (cardiac) coil at the National Cerebrovascular and Cardiovascular Center or Dokkyo Medical University Hospital, a 1.5-T MR imager (MAGNETOM Aera; Siemens Healthineers) with 18-channel (body) coils and 32-channel (spine) coils at the Ehime Prefectural Imabari Hospital, or a 1.5-T MR system (Achieva; Philips Healthcare, Best, the Netherlands) using a 32-element torso cardiac phased-array coil at the University of Tsukuba, Shin-Koga Hospital, Nihon University School of Medicine, or Kanagawa Cardiovascular and Respiratory Center. The procedures used to acquire CMR images at each institution are based on a standardized protocol (Supplementary Table 1).

### Core laboratory CMR analysis

All CMR images will be assessed by at least two trained observers who are unaware of patient data and used the T1W images to calculate PMR and CMRA images to evaluate vessel stenosis at the central core laboratory. The methods used to evaluate plaque images in this study have been described previously [6, 12]. In brief, in segment-based analysis, the highest signal intensity detected in each plaque will be considered the representative PMR value for that plaque. In patient-based analysis, the highest PMR among all coronary plaques is considered to be the PMR for that patient. Examination of coronary segments is limited to segments 1–3, 5–7, 9, and 11–13, based on recommendations from the American Heart Association [13].

The location of a coronary plaque is determined by careful comparison using fiduciary points on CMRA images. Once a coronary plaque has been confirmed with CMRA, the corresponding areas on T1W images were carefully matched using the surrounding cardiac and chest wall structures [6, 12, 14]. Regarding PMR quantification of coronary plaques that are not HIPs, when CMRA shows coronary stenosis in segments 1–3, 5–7, or 11–13, the PMR of the target lesion will be measured using the co-registration method described above.

### Definition of CAD

CAD is defined as a history of myocardial infarction, angina pectoris, PCI, CABG, or therapy with intracoronary fibrinolytic agents. In addition, patients will be classified based on CTCA or invasive coronary angiography showing the presence of the following for more than 1 year: (a) obstructive CAD if there is atherosclerotic plaque encompassing a luminal cross-sectional area of ≥ 70% in at least 1 major epicardial vessel or ≥ 50% in the left main trunk; (b) non-obstructive CAD if there is either atherosclerotic plaque encompassing a luminal cross-sectional area of < 70% but > 10% in at least 1 major epicardial vessel or a calcium score > 400 Hounsfield units; or (c) minimal or no CAD. Significant plaque burden is defined as atherosclerotic plaque causing > 10% luminal cross-sectional area stenosis.

### Blood tests

If the participant has not had levels of low-density lipoprotein cholesterol, high-density lipoprotein cholesterol, and triglycerides measured within the past 8 weeks, a blood test will be performed in the outpatient clinic. If total cholesterol is > 7.0 mmol/l or HDL is < 0.5 mmol/l, the primary care physician will be informed by letter, as this may warrant treatment irrespective of the patient’s 10-year CAD risk.

### Data analysis

The trial results will be reported in accordance with CONSORT guidelines. When possible, the clinical profile of non-recruited and ineligible patients will be recorded. All imaging and laboratory data were analyzed by an independent attending physician and a radiologist at the National Cerebral and Cardiovascular Center or Tohoku University. These evaluators were blinded to patient treatment status. This study was approved by the institutional review boards (IRBs) of the National Cerebral and Cardiovascular Center (study no. R21022-3) and 7 other participating institutions (Supplementary Figure 1). Written informed consent will be obtained from all enrolled patients.

### Statistical analysis

The study statistician (K. N.) will supervise statistical analyses performed by the Department of Preventive Medicine and Epidemiology, National Cerebral and Cardiovascular Center. A full statistical analysis plan will be prepared separately.

All analyses will perform according to the intention-to-treat principle. Missing data will be removed from the analyses, except for data on deaths, which are censored at the time the patient is lost from the trial. Endpoints will be analyzed with the use of Cox regression models, adjusted for center and minimization variables, and cumulative event curves will be constructed.

Data are reported as means and standard deviations, medians and interquartile ranges, and hazard ratios or odds ratios with 95% confidence intervals, as appropriate. All statistical analyses will be performed with SPSS software (version 24.0; IBM Corp, Armonk, NY), Stata 15 (StataCorp, College Station, TX), or R software (version 3.4.3; R Foundation for Statistical Computing). All *p* values < 0.05 will be considered statistically significant.

### Sample size

Based on the results of previous studies [6, 12, 14], we hypothesized that the 10-year probability of developing CAD would be ≥ 22% and that the effect of optimal drug therapy, such as a strong statin to reach the low-density lipoprotein (LDL) cholesterol control target and medications to reach the blood pressure control target, and the effect of screening with CMRA and non-contrast T1WI on patients with HIPs or coronary stenosis would result in 48–50% lower relative risk. The effect of early detection of coronary artery lesions (significant stenosis and unstable plaque) was estimated to result in a relative risk reduction of 48–50%. The SCOT-HEART study compared patients undergoing coronary CTCA versus conventional testing, similar to this study. The hazard ratio was 0.59 in the CTCA group compared with the conventional test group. We assumed that CTCA in the SCOT-HEART study [1, 15, 16] did not assess plaque instability and that CMR with analysis of HIPs could assess plaque instability better than CTCA alone. In a previous study [14], 77% of all cardiovascular events occurred within 2 years, and the event rate for HIP with signal intensity > 1.4 was approximately 30%; thus, an annual event rate of 11.6% was expected. Based on additional assumptions about an enrollment period of 3 years and a follow-up period of 3 years, power of 0.8, and two-sided *p* value of 0.05, a total of 524 patients would be needed with the dropout rate set at 5%.

### Randomization

In this study, dynamic allocation will be used to ensure even allocation of factors that might affect the primary endpoint. After a study investigator confirms the eligibility of the patient and obtains consent for participation, dynamic allocation will be performed by the study investigator with respect to age, gender, presence or absence of type II diabetes, and CKD grade. Dynamic allocation will be performed at the allocation center (Medical Edge, Tokyo, Japan). The allocation table and the most recent breakdown of allocated subjects in each group will be used. A web-based system will be used to automatically register and allocate subjects on a 24-h basis without any human operators. After confirming that a patient meets all the eligibility criteria, the study investigator will enter the necessary items into the web-based system for registration. If there are no problems, the system will allocate the patient using the randomized minimization method based on allocation adjustment factors. The results of the allocation will be shown automatically on the website. The study investigator will start the examinations based on the notification and record them on the case report form (CRF) for each participant.

The minimization method will be employed for assignment to the study treatment in this study. The ratio of the two groups to be adjusted will be 1:1 for the CMR group and the control group. The group to which the new cases will be assigned will be determined using the following procedure. The number of subjects assigned to the category to which the patient belongs for all allocation factors in the control and CMR groups will be simply summed. If the calculated total numbers for the two groups are equal, a random assignment will be performed with the same probability (1/2). If they are not equal, the patient will be assigned to the smaller group with a probability of 1.

### Allocation factors

Dynamic allocation is performed with respect to the following factors:

- Age at the time of consent
- Gender: male or female
- Presence or absence of type 2 diabetes mellitus
- CKD grade: 3a, 3b, 4, or 5

### Blinding

All stress myocardial scintigraphy and CMR measurements will be analyzed by an independent attending physician and a radiologist at the National Cerebral and Cardiovascular Center or in the Department of Diagnostic Radiology at Tohoku University Hospital. These evaluators will be blinded to patient allocation.

### Data quality control and management

The study investigator must complete a CRF for each participant who provides written informed consent. CRFs will be prepared in Japanese. Every institution of this study will create an institutional account for data entry and modification. The study representative or a person designated by the study representative will authorize access to the electronic CRF system for the study investigator. The completed CRFs are the property of the director of the study center. Personal information will be anonymized using a correspondence table with trial-specific identification numbers and stored in a locked room. Study investigators must not disclose the information contained in the CRFs to third parties without written approval from the director of the study center, except for regulatory agencies. Only investigators can access data. The datasets used or analyzed will be available from the principal investigator on reasonable request after the study has been completed. Data are collected via a CRF in an electronic data capture (EDC) system and stored and managed securely by the data monitoring committee.

### Study organization

The research group consists of researchers and statisticians at the National Cerebral and Cardiovascular Center in Suita Japan and at the Department of Diagnostic Radiology at Tohoku University Hospital in Sendai Japan and an independent data monitoring committee.

### Monitoring

The AQUAMARINE-CKD study will monitor safety by collecting adverse events (AEs) during the study period. An AE is defined as any medically unfavorable event that occurs in a study participant. An AE does not necessarily have to be causally related to the study. When an AE occurs, the principal investigator or study investigator must take immediate and appropriate action and follow up on all patients experiencing the AE until symptoms resolve or clinically significant laboratory abnormalities return to baseline. All AEs will be evaluated and documented on the CRF. The data monitoring committee is independent of the investigators. Committee members do not have competing interests. No interim analysis is planned.

### Health economic evaluation

A health economic evaluation will be performed to assess the cost-effectiveness of a diagnostic strategy using non-contrast T1WI compared with alternative diagnostic strategies for primary CAD prevention. A model-based cost-effectiveness analysis will be conducted to predict healthcare costs and quality-adjusted life years (QALYs) associated with a diagnostic strategy using non-contrast T1WI versus alternative strategies. The model consists of a short-term (within-trial) phase and a long-term phase. In the short-term decision model, study participants will be screened using each diagnostic strategy and will receive treatment if coronary HIPs are present. A long-term state transition model of mortality and cardiac events will be developed to extrapolate within-trial data. Parameter inputs such as risk, treatment effect, cost, and utility will be estimated using patient-level trial data or the best available data sources. In this economic evaluation, we will consider only direct medical costs from the perspective of the Japanese healthcare system. A lifetime horizon and an annual discount rate of 2% for costs and effectiveness will be applied. The incremental cost-effectiveness ratio (ICER) will be estimated using the following formula: ICER = (cost of non-contrast T1WI − cost of alternatives)/(QALY of non-contrast T1WI − QALY of alternatives). Deterministic and probabilistic sensitivity analyses will be performed to assess the influence of parameter uncertainty on base case results. Probabilistic sensitivity analyses will be performed using 1000 iterations of a second-order Monte Carlo simulation. Based on probabilistic sensitivity analyses, we will construct cost-effectiveness acceptability curves and estimate the proportion of iterations in which the diagnostic strategy with non-contrast T1WI would be preferred based on cost-effectiveness, assuming a willingness to pay a threshold of JPY 5,000,000 per QALY gained. We will also perform subgroup analyses with different combinations of baseline risks and treatment effects.

## Discussion

This prospective multicenter randomized study will assess the added value of the clinical implementation of non-contrast T1WI of coronary plaques and CMRA in an outcome-focused care pathway for patients at high risk for CAD. This study will help define the most appropriate use of this emerging technology for diagnosing and treating patients at high risk for CAD.

The role of diagnostic coronary artery testing in triaging patients with stable CAD continues to evolve towards recognizing the benefits of CTCA over functional testing. On the basis of findings from the landmark SCOT-HEART trial [1, 2, 17] evaluating the role of CTCA in patients with suspected stable CAD, anatomical testing has been proposed. Hence, the recent National Institute for Health and Care Excellence guidelines now recommend CTCA as the first-line method for the investigation of anginal chest pain in patients without known CAD [18].

On the other hand, coronary CMR plaque imaging can identify high-risk coronary plaques by exploiting the T1 shortening effects of plaque features (intraplaque hemorrhage, thrombus, lipid core, presence of macrophages, and cholesterol clusters), with or without exogenous contrast agents [19–23]. CMR identification of high-risk plaque is prognostically significant, independent of coronary luminal stenosis [6]. However, the clinical usefulness of this technique as primary prevention for CAD has not been elucidated. Therefore, this prospective study will provide novel insights on the effect of non-contrast T1WI in triaging high-risk patients with stable CAD.

Economic evaluation will assist policymakers in deciding whether CMR is cost-effective. CMR is an expensive technology, and its healthcare value needs to be established. Potential benefits of CMR might include reducing further noninvasive and invasive investigations and improving long-term clinical outcomes. Thus, evaluation of cost-effectiveness requires estimates of resource utilization, quality of life for all patients, and, in a subsequent follow-up study, event-free survival. The utility of CMR in patients with CAD is currently undefined. We believe that a randomized controlled trial is required to evaluate this emerging and promising imaging technology in a comprehensive and pragmatic manner.

## Trial status

Protocol version number and date: version 1: August 11, 2021

Date when recruitment began: September 10, 2021

Approximate date when recruitment will be complete: March 31, 2024

## Supplementary Information

**Additional file 1: Supplementary Figure 1.** Participating clinical research institutions.

**Additional file 2: Supplementary Table 1.** Standardized protocol for non-contrast T1-weighted imaging to assess coronary plaques: recommended protocol for CMR coronary plaque imaging.

## Data Availability

The datasets used and/or analyzed will be available from the corresponding author on reasonable request after the study is complete.

## Abbreviations

CAD: Coronary artery disease
CMR: Cardiac magnetic resonance
CMRA: Coronary magnetic resonance angiography
ECG: Electrocardiography
HDL: High-density lipoprotein
HIP: High-intensity plaque
LDL: Low-density lipoprotein
MI: Myocardial infarction
PCI: Percutaneous coronary intervention
CABG: Coronary artery bypass grafting
PMR: Plaque-to-myocardium signal intensity ratio
T1WI: T1-weighted imaging
TG: Triglycerides
