# Supplementary figures and images for: Clinical impact of cardiac magnetic resonance in patients with suspected coronary artery disease associated with chronic kidney disease (AQUAMARINE-CKD study): study protocol for a randomized controlled trial
Source: Trials. 2022 Oct 24;23:904. doi: 10.1186/s13063-022-06820-w (PMC9590223; doi:10.1186/s13063-022-06820-w)

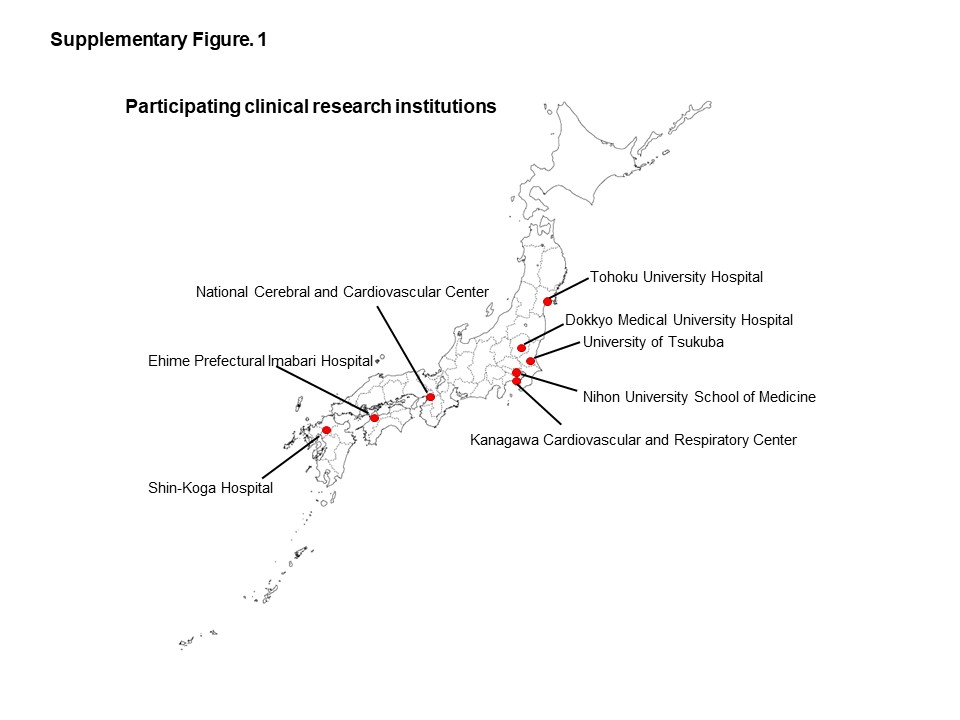

Supplement: Supplementary file 1 — Additional file 1: Supplementary Figure 1. Participating clinical research institutions. [file 13063_2022_6820_MOESM1_ESM.jpg]
