# Supplementary material for: Clinical impact of cardiac magnetic resonance in patients with suspected coronary artery disease associated with chronic kidney disease (AQUAMARINE-CKD study): study protocol for a randomized controlled trial
Source: Trials. 2022 Oct 24;23:904. doi: 10.1186/s13063-022-06820-w (PMC9590223; doi:10.1186/s13063-022-06820-w)
Supplement: Supplementary file 2 — Additional file 2: Supplementary Table 1. Standardized protocol for non-contrast T1-weighted imaging to assess coronary plaques: recommended protocol for CMR coronary plaque imaging. [file 13063_2022_6820_MOESM2_ESM.docx]

**Supplementary Table 1**

**Standardized protocol for non-contrast T1-weighted imaging to assess coronary plaques: recommended protocol for CMR coronary plaque imaging**

**MRI machine used**

- Siemens AG Healthcare Sector 1.5 or 3 Tesla
- Philips Medical Systems 1.5 or 3 Tesla

**Heart rate at the time of imaging**

- When heart rate is expected to exceed 75 beats per minute (bpm), adjust with administration of metoprolol (20 or 40 mg) 60 minutes before imaging. If the pre-imaging heart rate is less than 75 bpm, the patient is eligible for examination.
- Nitroglycerin (0.3 mg) can also be administered sublingually immediately before imaging to obtain high-quality MR images.

**Determination of coronary quiescence time**

- Focus survey images around the heart. Obtain reference images with free breathing to improve the sensitivity of parallel imaging.
- Obtain cine-MR images in a cross-sectional view of right coronary artery segments 1–2 in the short axis to determine coronary artery resting time. Determine data acquisition duration (data window duration) and trigger delay time (data window start) accordingly.

**Respiratory synchronization**

- Perform imaging with resting breathing. Set the navigator echo for respiratory synchronization at the highest point of the right diaphragm. Collect data during maximal expiration.

**Non-contrast T1-weighted imaging conditions**

- Set the cross-sectional image as a “transverse image” and the area to include the whole heart.
- Imaging using inversion recovery-based gradient echo: imaging with IR-based GRE.

**Recommended 3T-MRI Parameters**

・T1-weighted imaging (IR-based GRE)

Number of slabs: 1, Orientation: transverse, Phase encode direction: AP, Slices per slab: 130 (increase or decrease as appropriate), FoV: 280 mm x 266 mm,

Acquired slice thickness: 1.2 mm, Reconstruction slice thickness: 0.6 mm,

TR: 3.2 ms, TE: 1.4 ms, Averages: 2, Magnetization preparation pulse non-slice selective inversion recovery (IR) pulse, TI: 650 ms, Flip angle: 15 deg, Fat suppression: SPIR, Acquisition matrix: 232x200, Reconstruction matrix: 480, Trajectory: Cartesian, SENSitivity Encoding (SENSE), Acceleration factor: 2, Triggering: 1 RR cycle, Data acquisition duration: 80–150 ms (determined by coronary artery resting time), Trigger delay time (determined by coronary artery resting time), TFE factor: 31 (determined by coronary artery resting time), Navigator technique: On, Acceptance window: ± 2.0 mm, Navigator tracking factor: 0.60, Bandwidth: 1146.3 Hz

- Whole heart coronary angiography (3D-FFE)

Number of slabs: 1, Orientation: transverse, Phase encode direction: AP, Slices per slab: 150 (increase or decrease as appropriate), FoV: 300 mm x 281 mm,

Acquired slice thickness: 1.6 mm, Reconstruction slice thickness: 0.8 mm,

TR: 4.3 ms, TE: 1.4/2.6 ms, Averages: 2, Magnetization preparation pulse T2 prep: 60 ms, Flip angle: 15 deg, Fat suppression: mDixon, Acquisition matrix: 252x233, Reconstruction matrix: 512, Trajectory: Cartesian, CS-SENSE Acceleration factor: 6, Triggering: 1 RR cycle, Data acquisition duration: 80–150 ms (determined by coronary artery resting time), Trigger delay time: (determined by coronary artery resting time), TFE factor: 16 (determined by coronary artery resting time), Navigator technique: On, Acceptance window: ± 2.0 mm, Navigator tracking factor: 0.60, Bandwidth: 1078.3 Hz

**Recommended 1.5T-MRI Parameters**

Number of slabs: 1, Orientation: transverse, Phase encode direction: AP, Phase oversampling: 0%, Slice oversampling: 38%, Slices per slab: 104 (increase or decrease as appropriate), FoV read: 280 mm, FoV phase: 81.3%, Reconstruction slice thickness: 1.00 mm, TR: 4.0 ms, TE: 2.22 ms, Averages: 1, Magnetization preparation pulse: Non-slice selective IR pulse, TI: 500 ms, Flip angle: 12 deg, Fat suppression: 1-2-1 binomial water excitation, Base resolution: 256, Phase resolution: 90%, Slice resolution: 50%, Phase: partial Fourier off, Slice: partial Fourier off, Trajectory: Cartesian, In-plane interpolation: On, Triggering: 1 RR cycle, Data acquisition duration: 80–150 ms (determined by coronary artery resting time), Trigger delay time: (determined by coronary artery resting time), Number of segments: 23 (determined by coronary artery resting time), Navigator technique: On, Acceptance window: ± 3.0 mm, Navigator tracking factor: 0.60, Bandwidth: 420 Hz/Px, Echo spacing: 4.0 ms.
